# Effect of Maintained Early Silvicultural Treatments over Two Rotations and Climate Change on Early Growth of Radiata Pine Plantations

**DOI:** 10.3390/plants15182752

**Published:** 2026-09-09

**Authors:** Daniel Bozo, Rafael Rubilar, Matías Pincheira, Timothy J. Albaugh, Rachel Cook, Otávio C. Campoe, Óscar Jara

**Affiliations:** 1 Cooperativa de Productividad Forestal, Departamento de Silvicultura, Facultad de Ciencias Forestales, Universidad de Concepción, Concepción 4030555, Chile; dbozo@udec.cl (D.B.); ojara2016@udec.cl (Ó.J.); 2 Centro Nacional de Excelencia para la Industria de la Madera (CENAMAD)—ANID BASAL FB210015, Pontificia Universidad Católica de Chile, Santiago 7820436, Chile; 3 Forestal Mininco S.A., Avenida Alemania 751, Los Ángeles 4440000, Chile; matias.pincheira@cmpc.com; 4 Forest Productivity Cooperative, Virginia Tech Department of Forest Resources and Environmental Conservation, 228 Cheatham Hall, Blacksburg, VA 24061, USA; talbaugh@vt.edu; 5 Forest Productivity Cooperative, Department of Forestry and Environmental Resources, North Carolina State University, Raleigh, NC 27695, USA; rlcook@ncsu.edu; 6 Forest Productivity Cooperative, Departamento de Ciências Florestais, Universidade Federal de Lavras, Lavras 37200, MG, Brazil; otavio.campoe@ufla.br

**Keywords:** competing vegetation, weed control, drought stress, climate change, forest sustainability

## Abstract

Sustaining forest plantation productivity across successive rotations is increasingly challenging under changing environmental conditions. This study evaluated whether repeated establishment silviculture can maintain early second-rotation growth performance of *Pinus radiata* D. Don in central-south volcanic sandy soils in Chile. An experiment encompassing soil preparation (shovel planting vs. subsoiling + bedding), fertilization (B vs. NPKB), and weed control (pre-planting only vs. 2-year banded application) was conducted over two rotations. Survival, diameter, height, and stand-volume responses were compared between rotations at age 3. The second-rotation establishment period received 33% less annual rainfall than the first. Across all treatment combinations, mean survival decreased from 90.5% during the first rotation to 62.2% during the second rotation, while mean stand volume decreased from 6.58 to 1.16 m^3^ ha^−1^. During the second rotation, weed control was the only treatment that significantly increased individual-tree and stand growth: plots receiving pre-planting weed control only averaged 38.7% survival and 0.29 m^3^ ha^−1^, whereas those receiving additional 2-year banded vegetation control averaged 85.8% survival and 2.02 m^3^ ha^−1^ stand volume. These findings indicate that vegetation control is key to sustaining *P. radiata* productivity. However, progressively unfavorable environmental conditions will jeopardize forest productivity across rotations.

## 1. Introduction

Forest plantations are increasingly expected to sustain wood production, carbon sequestration, and ecosystem services under more variable and stressful climatic conditions [1]. However, climate change is intensifying droughts, elevating temperatures, and increasing atmospheric water demand [2,3], with consequences for plantation establishment, survival, and productivity [1,4]. The establishment phase is particularly vulnerable because seedlings with shallow root systems depend strongly on near-surface soil water and nutrients and are highly susceptible to competing vegetation. Therefore, rapid early growth requires silvicultural practices tailored to site-specific limitations in water, nutrients, soil physical conditions, and interspecific competition [5,6,7].

*Pinus radiata* D. Don is one of the most important plantation species in the Southern Hemisphere, with extensive cultivation in Chile, New Zealand, Australia, South Africa, and Spain [8]. In Chile, *P. radiata* plantations have been established across a wide range of edaphoclimatic conditions, ranging from highly productive volcanic ash soils to dry sandy soils characterized by low fertility, coarse texture, rapid drainage, and limited water-holding capacity. Across Chilean *P. radiata* plantations, water availability—determined mainly by precipitation and soil water-holding capacity—and potential evapotranspiration have been identified as major drivers of leaf-area development and volume growth [9,10]. Consequently, seedlings planted in sandy soils face elevated mortality risks during establishment, particularly under conditions of diminished rainfall and/or increased atmospheric water demand.

Given these challenges, establishment silviculture provides the primary tools to mitigate early resource limitations and accelerate site occupancy. Soil preparation can improve root exploration and reduce physical barriers, fertilization can alleviate nutrient deficiencies, and weed control reduces competition for water, nutrients, light, and growing space [5,6,7]. However, treatment responses are strongly site-dependent. Although soil preparation and fertilization can increase early growth by enhancing access to belowground resources, their benefits may be weak, transient, or contingent on the control of competing vegetation [5]. Weed control is often decisive at water-limited sites because competing vegetation can rapidly capture shallow soil water and nutrients when planted seedlings are most vulnerable [6,11,12].

To investigate these dynamics, the Chilean RW7 experiment was established as part of a network of long-term silviculture trials developed by the Forest Productivity Cooperative. During the first rotation, Rubilar et al. [5] evaluated these treatments across dry sand, red clay, and recent volcanic ash soils. The dry-sand site from their evaluation is the same location used in the present study. In the previous study at this site, weed control produced the most sustained gains through harvest age, whereas fertilization had comparatively little effect, and early responses to subsoiling diminished over the rotation [5]. These treatment responses over the 22-year first rotation, from establishment in 2000 to harvest in 2022, suggest that interspecific competition and water limitation were more important constraints on productivity at this sandy site than soil physical limitations alone.

Maintaining productivity across successive rotations is a major challenge for plantation forestry because harvesting operations, residue management, soil disturbance, nutrient export, and changes in organic matter inputs can alter resource availability for the subsequent rotation [13,14,15]. Nevertheless, successive-rotation experiments that maintain the same experimental plots, factorial structure, and treatment combinations are rare, making it difficult to determine whether effective establishment practices remain beneficial when repeated following harvest under different environmental conditions. The main innovation of this study is the maintenance of the same experimental units and factorial treatment structure across two successive rotations. This experimental continuity enables a rare direct comparison of early treatment responses between rotations at the same site under contrasting establishment conditions, complementing climate-growth assessments conducted within a single rotation or across different sites.

Our central research question is whether the treatment performance observed during the first rotation at the dry-sand site is maintained when treatments are reapplied after harvest. To address this, the specific objectives of this study are: (i) to determine whether repeated soil preparation, fertilization, and weed control affect the early survival and growth of second-rotation *P. radiata* under the environmental conditions observed during establishment; (ii) to ascertain whether treatments that reduce competition for water and nutrients maintain early stand performance across rotations and ameliorate the effects of any reduction in water availability; and (iii) to investigate whether soil preparation and fertilization improve early performance when combined with weed control. We hypothesized that, if water availability during second-rotation establishment was lower than during the first rotation, survival and growth would be reduced across all treatment combinations. However, we predicted that treatments incorporating weed control would exhibit smaller declines due to their capacity to reduce interspecific competition for limited soil water and nutrients. We also hypothesized that fertilization would produce positive responses primarily when combined with weed control. Conversely, we expected that subsoiling would have weak or null effects, as increasing soil disturbance alone would not overcome the dominant limitation imposed by low water availability on this coarse-textured sandy soil.

## 2. Results

### 2.1. Climatic Conditions During Early Establishment Between Rotations

Climatic conditions during stand establishment varied substantially between the first (2000–2003) and second (2022–2025) rotations (Table 1). Mean annual precipitation (Pp) declined by 33%, dropping from 1337 mm yr^−1^ during the first rotation to 898 mm yr^−1^ during the second rotation. Mean growing-season precipitation (PpGs) decreased from 508 mm during the first rotation to 288 mm during the second rotation, corresponding to a reduction of 43%. Conversely, mean annual potential evapotranspiration (PET) increased from 1077 to 1153 mm yr^−1^.

**Figure 1:**
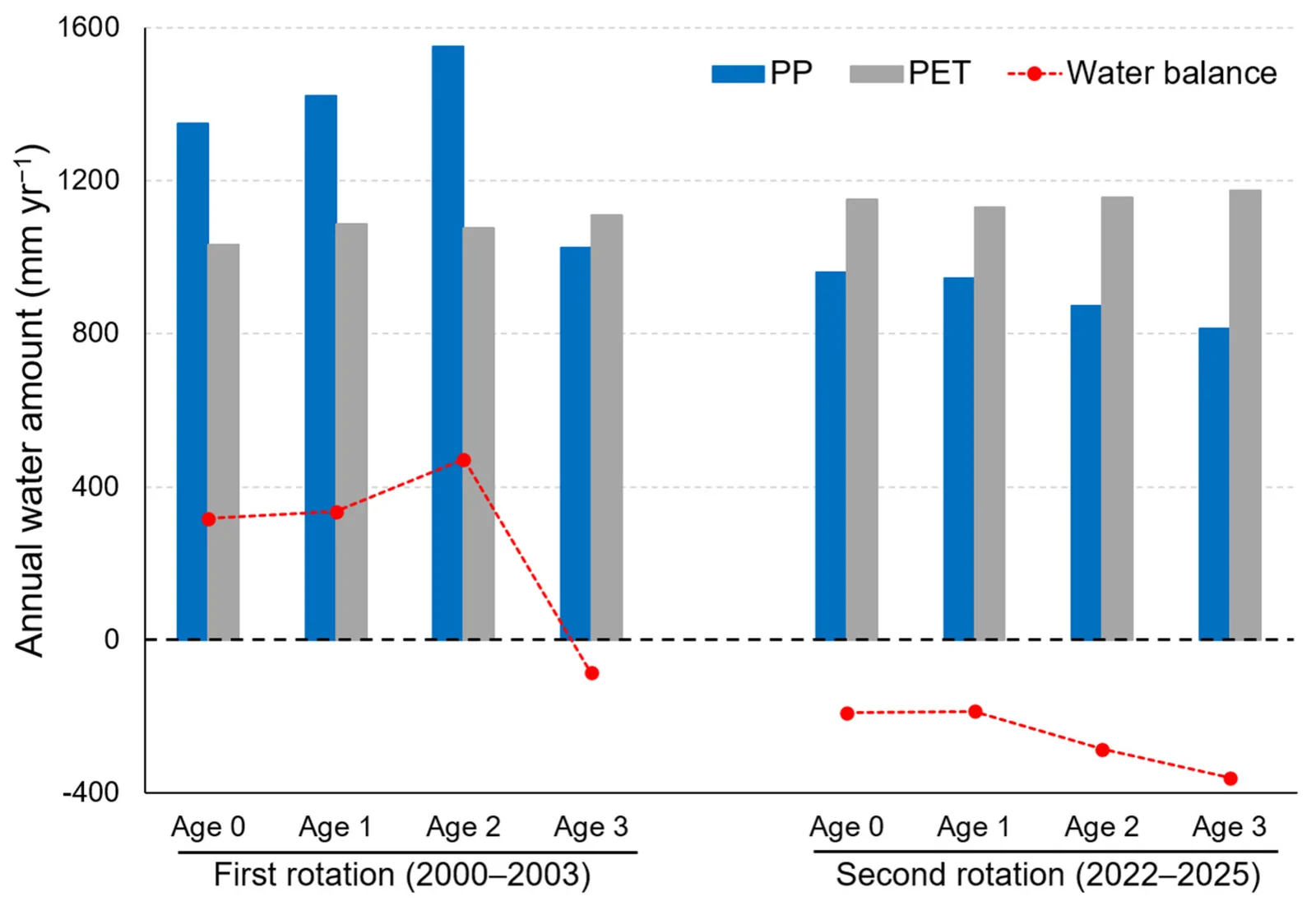
Annual precipitation (Pp), potential evapotranspiration (PET), and climatic water balance during the first four years after planting in the first and second rotations. Water balance was calculated as Pp—PET.

Consequently, the overall water balance shifted from positive to negative between rotations (Figure 1). During the first rotation, precipitation surpassed atmospheric water demand, resulting in a positive mean annual water balance (+260 mm yr^−1^) and an aridity index (AI) of 1.25. In contrast, the second rotation was characterized by a water deficit (−255 mm yr^−1^) and a lower AI of 0.78, confirming substantially drier establishment conditions.

Temperature-related indicators also reflected a more stressful climatic environment during the second rotation. The mean annual vapor pressure deficit (VPD) increased from 0.71 to 0.81 kPa, while the frequency of extreme temperature events rose markedly. The annual number of days with maximum temperatures exceeding 35 °C climbed from 2.3 to 6.3 days yr^−1^ (a 174% increase). Likewise, the frequency of frost days grew from 25 to 42 days yr^−1^, representing a 68% increase between the two rotations.

Overall, these climatic analyses reveal a clear shift from relatively humid establishment conditions during the first rotation to substantially drier and more stressful conditions during the second rotation. This change is characterized by a decline in precipitation, an increase in atmospheric water demand, and a heightened frequency of temperature extremes.

### 2.2. Survival and Early Growth Responses

At age 3, survival and early growth varied significantly among establishment treatments within each rotation (Table 2 and Table 3). During the first rotation, the control treatment (S0F0WC0) attained 75.0% survival, 3.6 cm diameter, 1.5 m height, and 1.9 m^3^ ha^−1^ stand volume. The most significant increases were observed in treatments involving weed control (WC1), where stand volume ranged from 8.8 to 13.8 m^3^ ha^−1^, corresponding to relative gains of 355% to 612% over the control. The highest first-rotation performance was recorded in S1F1WC1, which achieved 99.5% survival, 6.5 cm diameter, 2.5 m height, and 13.8 m^3^ ha^−1^ stand volume. During the second rotation, absolute survival and growth were diminished, but the treatment pattern emerged more clearly. The control yielded only 37.0% survival, 2.4 cm diameter, 1.0 m height, and 0.3 m^3^ ha^−1^ stand volume. Treatments without weed control (WC0) showed similarly low survival (34.8% to 46.3%) and stand volume (0.2 to 0.4 m^3^ ha^−1^). Conversely, treatments including weed control (WC1) attained 83.0% to 89.8% survival and 1.6 to 2.5 m^3^ ha^−1^ stand volume. The greatest second-rotation volume response was observed for S0F1WC1, which reached 2.5 m^3^ ha^−1^, a 788% increase over the control.

During the first rotation, soil preparation significantly affected survival and stand volume, whereas fertilization significantly affected all growth metrics except survival (Table 3). Weed control universally improved all variables, and significant interactions occurred for S × WC on survival and F × WC on stand volume. During the second rotation, treatment dynamics simplified, establishing weed control as the predominant treatment factor. Weed control alone significantly affected all variables (Table 2 and Table 3), while fertilization affected height only, and soil preparation and interactions were non-significant. Overall, these results indicate that early second-rotation establishment success in this sandy soil was predominantly determined by weed control rather than by soil preparation or fertilization.

At the main factor level, weed control (WC) yielded the most substantial and consistent gains in both rotations. During the first rotation, WC increased diameter (3.8 to 6.0 cm), height (1.58 to 2.30 m), stand volume (2.52 to 10.64 m^3^ ha^−1^), and survival (82.1% to 99.0%). During the second rotation, WC similarly improved diameter (2.3 to 3.5 cm), height (0.9 to 1.4 m), stand volume (0.29 to 2.02 m^3^ ha^−1^), and survival (38.7% to 85.8%). Consequently, while absolute stand volume was lower during the second rotation, the relative importance of weed control increased, particularly for survival and stand volume. Soil preparation (S) and fertilization (F) produced weaker and less consistent responses than those of weed control (WC). During the first rotation, subsoiling increased stand volume from 5.9 to 7.3 m^3^ ha^−1^ and survival from 85.1% to 96.0%. However, during the second rotation, subsoiling did not improve early growth, with stand volume decreasing from 1.2 to 1.1 m^3^ ha^−1^ and diameter from 3.0 to 2.8 cm. Fertilization increased stand volume in the first (5.68 to 7.48 m^3^ ha^−1^) and second (0.9 to 1.4 m^3^ ha^−1^) rotations, but its effect was consistently smaller than that of weed control.

### 2.3. Comparison Between First and Second Rotations at Age 3

Across all treatment combinations, early stand performance was substantially lower during the second rotation than during the first rotation at age 3. Mean survival decreased from 90.5% to 62.2% (−31.3%). Similarly, mean diameter declined from 4.9 to 2.9 cm (−40.7%), height from 1.9 to 1.2 m (−39.7%), and stand volume from 6.58 to 1.16 m^3^ ha^−1^ (−82.4%).

The combined repeated-measures analysis confirmed a significant main effect of rotation on survival, diameter, height, and stand volume (all *p* < 0.001; Table 3). Significant rotation × weed control interactions were also detected for all response variables (all *p* < 0.001), demonstrating that the magnitude of the weed-control response differed between rotations. Weed control increased survival by 16.9 percentage points during the first rotation and by 47.1 percentage points during the second rotation. Similarly, stand volume increased from 2.52 to 10.64 m^3^ ha^−1^ during the first rotation and from 0.29 to 2.02 m^3^ ha^−1^ during the second rotation, representing relative increases of approximately 322% and 597%, respectively. A significant rotation × soil preparation × weed control interaction was also detected for survival (*p* = 0.011), indicating that the rotation-dependent survival response to weed control varied with soil preparation. Neither rotation × soil preparation nor rotation × fertilization was significant for any response variable, and all remaining interactions involving rotation were also non-significant (*p* > 0.05).

Stand volume decreased across all treatment combinations, with relative reductions ranging from 76.6% (S0F1WC1) to 94.5% (S1F0WC0). Survival also declined across all treatments, but the magnitude depended heavily on weed control. Treatments without weed control (WC0) showed survival reductions of 48.7% to 60.6%, while treatments with weed control (WC1) restricted these losses to just 9.3% to 16.4%.

**Figure 2:**
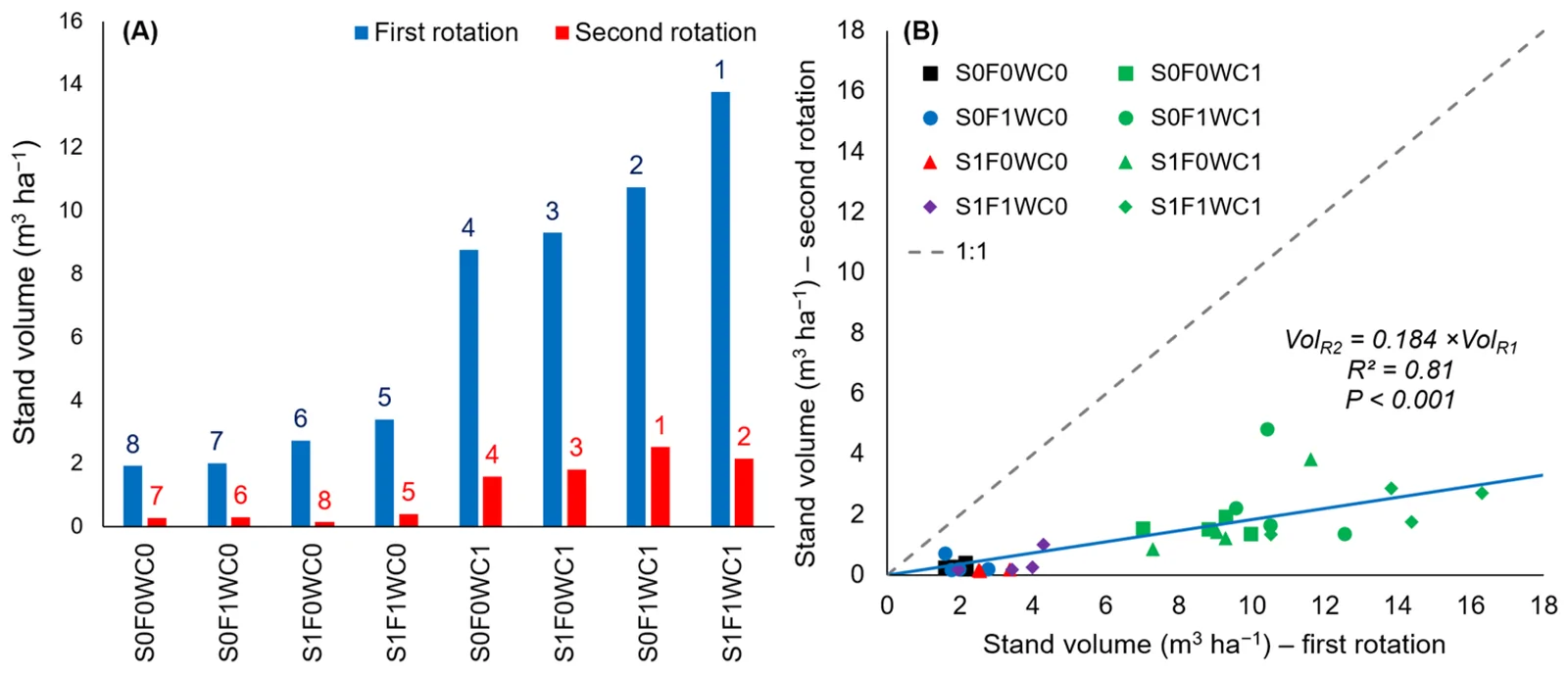
Rotation-to-rotation changes in stand volume and treatment ranking at age 3. Treatment codes indicate factorial treatment identities maintained across rotations. (**A**) Stand volume by treatment for both rotations; bars are ordered according to first-rotation stand volume. Numbers above bars indicate within-rotation ranking, where 1 = highest and 8 = lowest. (**B**) Relationship between first- and second-rotation stand volume across treatments using paired block-level observations. The gray dashed line represents the 1:1 relationship, and the solid blue line represents the regression model fitted through the origin.

Treatment ranking based on stand volume varied between rotations, but the separation driven by weed control remained constant (Figure 2A). During the first rotation, the treatment with the highest stand volume was S1F1WC1, followed by S0F1WC1, S1F0WC1, and S0F0WC1. During the second rotation, S0F1WC1 led, followed by S1F1WC1, S1F0WC1, and S0F0WC1. Thus, weed-control treatments consistently occupied the top four rankings across both rotations, leaving WC0 treatments at the bottom.

Second-rotation stand volume was strongly related to first-rotation stand volume across treatments (slope = 0.18; R^2^ = 0.81, *p* < 0.001; Figure 2B). This indicates that the second rotation attained approximately 18.4% of the first-rotation stand volume at age 3, equivalent to an overall reduction of 81.6%. Although all observations fell well below the 1:1 line, the relationship suggests that treatment performance was partially maintained across rotations, despite the substantial decline in early stand volume.

Overall, despite a strong rotation-to-rotation decrease in early growth and survival, the relative efficacy of weed control was maintained. Even though the best-performing treatment changed from S1F1WC1 (first rotation) to S0F1WC1 (second rotation), all top-tier treatments included weed control. This suggests that weed control reliably ensures enhanced early stand performance across rotations.

## 3. Discussion

### 3.1. Early Productivity Across Contrasting Establishment Periods

This study offers a rare successive-rotation evaluation of repeated establishment silviculture in *Pinus radiata*, employing the same factorial treatment structure, plot design, and treatment identities post-harvest of the first rotation. This experimental continuity enabled us to assess whether the treatment performance observed during the first rotation at the dry-sand site was maintained when reapplied under different establishment conditions. Our results generally supported our hypotheses. As anticipated, early second-rotation survival and growth declined substantially compared to the first rotation, coinciding with a 33% reduction in mean annual precipitation, an increase in PET from 1077 to 1153 mm yr^−1^, and a shift in water balance from +260 to −255 mm yr^−1^. These changes were accompanied by a reduction in mean survival from 90.5% to 62.2% and in stand volume from 6.58 to 1.16 m^3^ ha^−1^ between rotations. This decline is consistent with broader evidence that warming, reduced water availability, and higher atmospheric water demand can reduce forest productivity by intensifying soil water deficits, increasing vapor pressure deficit, limiting stomatal conductance and carbon uptake, and increasing mortality risk [16,17,18]. Severe drought and heat events have been associated with marked reductions in terrestrial primary productivity and widespread tree mortality across forest biomes [18,19]. The efficacy of weed control aligned with the hypothesized responses, maintaining its position as the predominant treatment and the only practice that significantly increased all growth response variables during the second rotation. Conversely, fertilization yielded only marginal support for our hypothesis, improving performance primarily when employed in conjunction with weed control; however, it was insufficient to offset the absence of competing vegetation control. Finally, the weak or null response to subsoiling during the second rotation supported our hypothesis that deep soil disturbance alone cannot overcome the dominant limitations imposed by lower water availability and early resource competition on this coarse-textured sandy soil.

Climate change is reshaping the environmental conditions under which forest plantations are established and managed, particularly through increasing temperatures, higher atmospheric water demand, and more frequent or severe droughts [1,4]. These changes are particularly relevant in Mediterranean-type and temperate regions, where projected reductions in precipitation and increases in evaporative demand may intensify seasonal water deficit during the growing season [1,20]. Similar to other parts of the world, radiata pine plantations in Chile are limited by water availability, which has been identified as a significant factor influencing leaf-area development, growth efficiency, and stand productivity, with lower water deficits generally associated with greater leaf area and volume growth [9,10]. These climatic constraints are especially critical because planted seedlings, which have small root systems, depend strongly on near-surface soil water and compete directly with non-planted vegetation for water, nutrients, light, and growing space [6,11,12]. By comparing two establishment periods on the same experimental site, our study establishes a link between broader climatic concerns and an operational question: which establishment practices remain effective when the same sandy site is replanted under drier conditions? In this context, the second rotation evaluated in our study can be interpreted as an operational example of establishment under climatic conditions consistent with projected trends: lower precipitation, higher PET, and a more negative water balance (Table 1). The substantial decline in early survival and stand volume documented in this study (Table 2) indicates that water-limited sandy sites may become increasingly vulnerable unless establishment practices are modified to reduce early competition for scarce water resources. Therefore, sustaining plantation productivity under increasingly water-limited conditions will depend on identifying establishment practices that enhance early survival, expedite site occupancy, and mitigate competition for scarce resources [5,6].

### 3.2. Silvicultural Treatment Responses Across Rotations

Under this more restrictive climatic context, weed control emerged as the primary driver of early stand establishment. As shown by the severe mortality in WC0 plots, early competition in water-limited sandy soils is a central determinant of site capture. During the initial years, young trees are restricted to shallow soil layers, making the competition for near-surface water particularly intense. Studies on *P. radiata* have consistently demonstrated that failing to manage this competing vegetation reduces early growth by limiting water and nutrient availability, with negative effects that persist long beyond the initial establishment phase [6,11,12].

The significant rotation × weed control interactions further refine this interpretation by demonstrating that the benefit of weed control was maintained across rotations, although its magnitude was not constant. Its positive effect on survival was substantially greater during the second rotation, consistent with stronger competition for limited resources under the more restrictive establishment conditions. Although the absolute weed-control gains in diameter, height, and stand volume were smaller during the second rotation because overall growth was strongly reduced, weed control produced a greater proportional increase in stand volume relative to WC0 plots. The significant rotation × soil preparation × weed control interaction for survival further indicates that the change in the survival response to weed control depended partly on soil preparation. However, because this higher-order interaction was not significant for the growth variables and the rotation × soil preparation interaction was consistently non-significant, it should be interpreted as a survival-specific response rather than evidence of a generalized benefit of subsoiling. Likewise, the non-significant rotation × fertilization interactions indicate that there was no statistically demonstrated change in the fertilizer response between the two establishment periods, although fertilizer formulations and doses differed between rotations.

The findings of the present study are particularly relevant because they are consistent with the long-term treatment performance and results previously observed at the same dry-sand site. Rubilar et al. [5] showed that, at rotation age, weed control produced the most significant and persistent productivity gain at this dry-sand site. Weed control led to an increase in cumulative volume of approximately 56 m^3^ ha^−1^ (20% gain). In contrast, fertilization exhibited a minimal response, while subsoiling presented null or negative effects. The results of our second-rotation experiment suggest that this treatment performance was repeated during the initial phase following replanting. The four treatments with the highest rankings in both rotations included plots with weed control, while treatments without weed control consistently occupied the lowest rankings. This continuity across temporal scales emerges as a pronounced finding of this study. It suggests that weed control is not merely an expediter of early growth; rather, it is a silvicultural practice that addresses a persistent site limitation. In other words, the same treatment component that explained long-term productivity gains through harvest age during the first rotation also explained early survival and growth during the second rotation. This strengthens the evidence indicating that water availability and interspecific competition, rather than soil physical limitations alone, serve as the primary determinants of early productivity potential on this sandy site.

The weak response to subsoiling further reinforces this interpretation. While soil preparation initially improved growth during the first rotation, its effect completely disappeared and even trended negative under the drier conditions of the second rotation. This aligns well with Rubilar et al. [5], who noted that subsoiling responses can diminish over time and become null or negative across different soil-site conditions. The most probable explanation is that subsoiling did not address the predominant constraint at this site. In coarse-textured sandy soils characterized by excessive drainage [21], water availability may have been more limiting than mechanical impedance under the conditions evaluated. Consequently, while deep soil disturbance has the potential to enhance root exploration, under conditions of diminished precipitation, elevated PET, and greater atmospheric water demand, soil loosening does not necessarily translate to an increase in plant-available water, particularly in excessively drained sandy soils [22,23]. This interpretation aligns with findings showing that reduced precipitation leads to an increased reliance on stored and deep soil water to maintain transpiration and productivity under drought conditions in pine plantations [24]. Furthermore, in the absence of effective weed control, the reduction in soil resistance caused by subsoiling may benefit competing vegetation as well as crop trees, potentially increasing competition for limited soil water and nutrients. A similar pattern of inconsistent responses to soil preparation has been documented in other pine systems, where early benefits disappeared or became negative depending on soil compaction and site resource limitations [25,26].

Fertilization exhibited an intermediate, highly conditional response. Fertilized treatments tended to show better early performance mainly when accompanied by weed control, whereas fertilization alone produced weak responses, indicating that nutrient addition could not compensate for the dominant limitation imposed by competition for water. However, fertilizer prescriptions differed substantially between rotations. During the first rotation, F1 supplied 29.5 g N and 32.4 g P plant^−1^ at establishment and included a second fertilizer application two years later. During the second rotation, F1 used the current operational controlled-release NPKB formulation, supplying 2.5 g N, 3.2 g P, 2.9 g K, and 0.25 g B plant^−1^. Differences in formulation, dose, and application regimen may have contributed to the weaker second-rotation response and should therefore be considered when comparing fertilizer effects between rotations. Nevertheless, the observed pattern is consistent with findings that competing vegetation can reduce fertilizer capture in young *P. radiata* plantations [12], and that fertilizer efficacy depends strongly on concurrent weed control [27]. Thus, additional nutrients without weed control can be largely taken up by non-crop vegetation, thereby increasing competitive pressure rather than fostering tree growth. Operationally, fertilization without weed control may therefore produce variable or less predictable responses, because the balance between nutrient uptake by crop trees and competing vegetation will determine whether the added resources translate into greater tree growth.

### 3.3. Management Implications, Limitations, and Future Monitoring

The decline in productivity during the second rotation indicates that the establishment practices evaluated here were unable to maintain first-rotation productivity levels under markedly different environmental conditions. Although treatment rankings were partly preserved, absolute performance declined across all treatment combinations, with the largest reductions occurring in the absence of weed control. Treatments including weed control maintained high survival rates and remained among the top-ranked treatments, despite producing substantially lower stand volume than during the first rotation. This suggests that weed control was the most effective practice for preserving relative tree growth performance under drier establishment conditions, but it was not sufficient to fully offset the reduction in water availability. The strong relationship between the first- and second-rotation stand volume further indicates that treatment effects were partially maintained across rotations, even though all treatments fell substantially below the 1:1 line (Figure 2). These results highlight a limitation of relying on current establishment systems to sustain productivity when environmental conditions change substantially between rotations. Although competition control, site preparation, and improved planting material are central components of plantation productivity, their effectiveness remains constrained by site water availability and climatic conditions [1,28]. Consequently, productivity projections based primarily on historical growth relationships may overestimate future wood supply if they do not explicitly account for climate-driven shifts in site productivity, changes in water availability, and the limited capacity of current silvicultural systems to compensate for large environmental differences among rotations [1,20,28,29].

The implications of these early establishment responses extend beyond the short-term differences in tree size because survival, stand density, and rapid canopy development during establishment can influence resource capture and subsequent stand productivity [5,6]. In the present study, the substantial reduction in second-rotation stand volume indicates a pronounced early productivity gap between rotations. Although weed control maintained higher survival and stand volume than WC0, it was insufficient to restore first-rotation performance, and the fitted rotation-to-rotation relationship indicated that stand volume at age 3 during the second rotation corresponded to approximately 18% of that observed during the first rotation. Whether this difference persists, narrows, or expands will depend on subsequent stand development and future water availability, which strongly influence leaf-area development and plantation productivity in *P. radiata* [9,10]. Continued monitoring through canopy closure and rotation age is therefore required before concluding that the observed early difference represents a persistent loss of site productivity. Nevertheless, the present results indicate that vegetation management can partially mitigate early productivity losses by reducing resource competition but cannot fully compensate for markedly more restrictive water conditions [1].

Because establishment costs, wood prices, and rotation-age yields were not evaluated, the economic viability of the second rotation cannot yet be determined. However, the pronounced early volume deficit may delay the recovery of establishment costs, emphasizing the need to combine continued growth monitoring with a formal economic analysis before drawing conclusions about rotation-scale profitability. Several limitations should be considered when interpreting these results. Although the comparison of two establishment periods cannot by itself establish a long-term climatic trend, the drier conditions experienced during the second rotation occurred within a broader regional drying context. Long-term observations have documented a multidecadal decline in precipitation across central-southern Chile, while regional climate projections indicate that precipitation reductions and increasing water deficits are likely to continue during the twenty-first century [30,31]. Therefore, the conditions observed during the second rotation are consistent with the documented and projected regional drying trend, although short-term climatic variability may have contributed to the magnitude of the difference between establishment periods. An additional limitation is that planting stock differed between rotations: the first rotation used 1–0 bare-root cuttings, whereas the second used containerized rooted cuttings. Because comparable measurements of initial seedling size, biomass, root traits, and stress tolerance were unavailable, the contribution of planting stock to the differences between rotations could not be quantified. Therefore, the observed decline in survival and growth should be interpreted as being associated with contrasting establishment conditions, including lower water availability, rather than attributed exclusively to climatic differences.

Operationally, sustaining early productivity on drought-prone sandy soils across successive rotations requires prioritizing practices that reduce resource competition. Under the drier second-rotation conditions, weed control was the sole treatment that consistently maintained survival, stand volume, and relative performance; subsoiling and fertilization alone failed to offset growth declines. While limited to a single site and early development phase, this study provides a rare, highly controlled comparison of successive rotations. Continued monitoring is needed to determine if this early growth decline persists, if treatment rankings hold through canopy closure, and if productivity eventually converges with the first rotation. Ultimately, weed control should be recognized not only as a growth-promoting practice but also as an important adaptation strategy for sustaining plantation establishment and productivity under increasingly water-limited conditions.

## 4. Materials and Methods

### 4.1. Study Area

**Figure 3:**
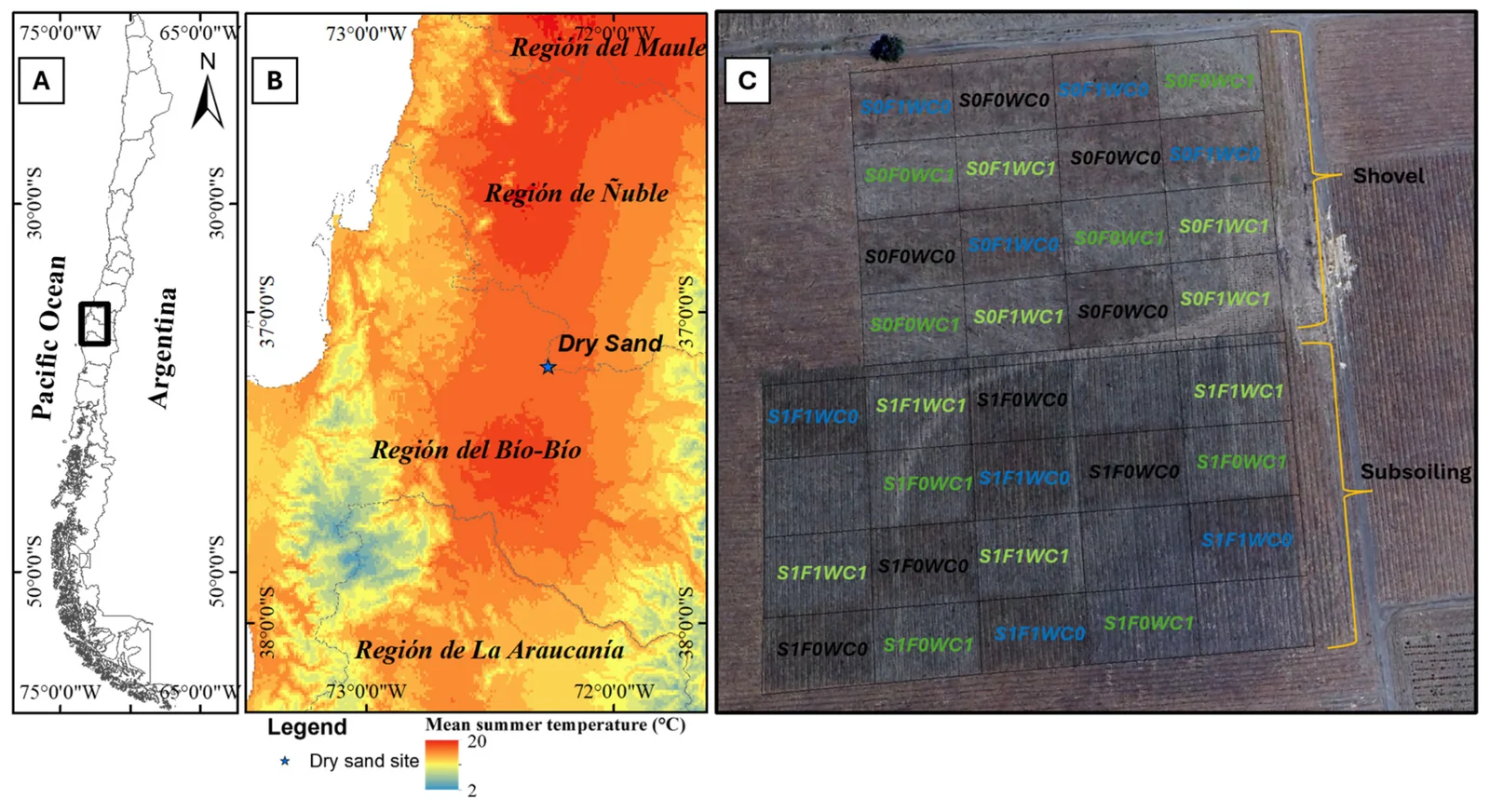
Location and experimental layout of the RW7 dry-sand site in south-central Chile. (**A**) Location of the study region within Chile. (**B**) Mean February temperature (°C) context and location of the dry-sand experimental site (blue star). (**C**) Aerial view of the 32 experimental plots approximately 1.5 years after establishment of the second rotation (November 2023), showing the spatial arrangement of soil preparation (S), fertilization (F), and weed control (WC) treatments. S0 and S1 represent shovel planting and mechanized soil preparation, respectively; F0 and F1 represent baseline and enhanced fertilization; and WC0 and WC1 represent baseline vegetation control and additional two-year banded weed control, respectively. The same plot boundaries and treatment identities were maintained across rotations, although the operational soil-preparation prescriptions differed.

This study was conducted at the dry-sand site of the Chilean RW7 network experiment, located in the northern Biobío Region of south-central Chile (37°10′40″ S, 72°15′47″ W) (Figure 3). The site features an andesitic–basaltic volcanic sandy soil, also known as the Arenales soil series [32], classified as fragmental, thermic Dystric Xerorthents. The soil has a coarse texture, containing 96–99% sand throughout the upper 1 m, which results in rapid water infiltration and excessive drainage. The plant-available water storage within the upper 1 m is only approximately 15 mm, indicating a very low water-holding capacity [21]. This coarse texture also contributes to low nutrient-retention capacity [32]. Furthermore, the soil exhibits a strong seasonal water limitation during the spring–summer growing period [5,21,32,33]. The climate at the site is characterized as Mediterranean-temperate, with precipitation concentrated mainly during winter months and a pronounced dry period in spring and summer. During the first rotation (2000–2022), the mean annual temperature at this site was 13.7 °C, and the mean annual precipitation ranged from 1135 to 1168 mm yr^−1^ [5].

### 4.2. Experimental Design

The RW7 Chile trial is part of a long-term experimental network established by the Forest Productivity Cooperative to evaluate how establishment silviculture modifies *P. radiata* productivity across contrasting soil physical, nutrient, and water limitations. The original experiment evaluated the individual and combined effects of soil preparation, fertilization, and weed control across an environmental gradient. A detailed description of the first-rotation experiment, site classification, and rotation-age treatment responses is provided by Rubilar et al. [5]. The first rotation at the dry-sand site was established in July 2000 and was harvested in May 2022 at 22 years of age. Following the harvest, the site was replanted in July 2022. The second rotation employed the same experimental layout, factorial treatment structure, plot boundaries, and experimental units as the first rotation.

The experimental design followed the original split-plot factorial structure of the RW7 Chile experiment described by Rubilar et al. [5] (Figure 3C). The trial comprised four blocks, with soil preparation (S) applied as the main-plot factor, and the factorial combination of fertilization (F) and weed control (WC) applied as subplot treatments within each soil-preparation main plot. Treatment plots encompassed an area of 0.4 ha, with an internal measurement plot of 100 trees. At the dry-sand site, trees were planted at a spacing of 4 × 2 m, which corresponds to an initial density of 1250 trees ha^−1^. Although the original factorial treatment identities, plot boundaries, and experimental units were maintained across rotations, some operational prescriptions differed between rotations to reflect contemporary establishment practices. Therefore, treatment applications for each rotation are described below.

The first rotation was established in July 2000 using full-sib, 1-0 bare-root cuttings. Soil preparation included shovel planting without deep mechanical soil disturbance (S0) and subsoiling to a depth of 80 cm followed by a 20 cm high bed before shovel planting (S1). Fertilization included boron-only (B) treatment (F0) and an enhanced fertilization of a complete fertilizer mixture comprising nitrogen (N), phosphorus (P), potassium (K), and boron (F1). In September 2000, all trees received 1.5 g B plant^−1^ because boron deficiency is common in soils of the study area [34]. Trees in the F1 plots additionally received 29.5 g N plant^−1^ and 32.4 g P plant^−1^ at establishment. The first-year fertilization was divided between two bands located 30 cm from each tree. A second fertilizer application was completed in September 2002 in the F1 plots, supplying 29.5 g N plant^−1^, 32.4 g P plant^−1^, 25.0 g K plant^−1^, and 3.0 g B plant^−1^. This second application was distributed along the planting row. Weed-control treatments included no weed control after establishment (WC0) and post-planting annual banded weed control until year 2 (WC1). In WC0, the development of competing vegetation was allowed post-planting. In WC1, weed control was applied in a 2 m wide band centered on the planting row during the first two years after planting. The chemical application consisted of glyphosate at 2 kg ha^−1^ and atrazine at 3 kg ha^−1^, in conjunction with Galactic surfactant at 1 mL L^−1^, applied during the first two growing seasons. This treatment was applied to reduce interspecific competition for soil water, nutrients, light, and growing space during the initial establishment of the stand.

The second rotation was established in July 2022 after harvesting, using containerized rooted cuttings. Thus, planting stock differed between rotations, reflecting the operational nursery material used at each establishment date. The original plot boundaries and S, F, and WC treatment identities were maintained; however, some operational prescriptions were adjusted to align with current establishment practices. S0 plots received no mechanical soil preparation beyond shovel planting, whereas soil preparation in S1 plots was conducted using a D6 bulldozer equipped with a disk harrow, maintaining the original soil preparation plot assignments. F0 plots received no NPKB fertilizer, whereas F1 received 25 g plant^−1^ of controlled-release NPKB fertilizer at establishment, corresponding to approximately 2.5 g N, 3.2 g P, 2.9 g K, and 0.25 g B plant^−1^ on an elemental basis. The fertilizer formulation and dose used during the second rotation were selected to reflect the controlled-release product currently applied in operational plantation establishment. To ensure that initial competing vegetation conditions were homogeneous across the 13.8 ha experimental area, a baseline post-planting herbicide application of simazine (3 kg ha^−1^) was administered in a 2 m band over the planting row across all plots. After this uniform baseline application, the weed-control treatments were differentiated according to the original assignment: WC0 plots received no additional operational vegetation control, while WC1 plots received additional banded control (2 m width) during the first two years after planting. During the establishment year, WC1 plots were subjected to an additional application in September, targeting mainly shrubs and woody competing vegetation. Thus, the factorial treatment identities were maintained across rotations, but the specific application products and nutrient doses differed from those utilized during the first rotation.

### 4.3. Stand Measurements and Calculated Variables

Tree survival and growth were evaluated during the establishment phase and summarized at age 3 to compare treatment performance within and between rotations. Survival was determined as the percentage of living trees relative to the initial number planted in each measurement plot. Root collar diameter (RCD, cm) and total height (H, m) were measured for all surviving trees. Because the trees were in the early establishment phase, their size was represented using a juvenile stem volume index derived from these RCD and height measurements. The individual-tree volume index (VI) was calculated using Equation (1):

(1) $$VI=\frac{{\pi \times (RCD/100)}^{2}\times H\times 0.33}{4},$$

where VI is the individual-tree volume index (m^3^ tree^−1^), RCD is the root collar diameter (cm), H is the total tree height (m), and 0.33 is the form factor utilized to approximate juvenile stem form. Plot-level stand volume was estimated by summing the individual-tree volumes for all living trees within each measurement plot and then scaling these values to a hectare basis.

Treatment means were calculated for diameter (RCD), total height, stand volume, and survival within each rotation and treatment combination. Absolute treatment responses were calculated relative to the control treatment (S0F0WC0) within each rotation using Equation (2):

*Response* = *Treatment mean* − *S0F0WC0 mean* (2)

Relative responses were calculated using Equation (3):

(3) $$Response\;\left(\%\right)=\frac{Treatment\;mean-S0F0WC0\;mean}{S0F0WC0\;mean}\times 100$$

### 4.4. Climatic Data and Water Balance

The daily climatic data for the first and second establishment periods were compiled using a multi-source approach to ensure temporal completeness and consistency. The first establishment period spanned 2000–2003, while the second spanned 2022–2025. Daily precipitation and air temperature were obtained primarily from the updated CR2MET gridded meteorological product for Chile available through 2025. CR2MET has a regular spatial resolution of 0.05° × 0.05°, equivalent to approximately 5 km in central Chile, and integrates ground observations, reanalysis, and topographic predictors [35]. Because the spatial resolution is coarser than the 13.8 ha experiment, CR2MET was used to characterize temporal differences in the climatic context between establishment periods rather than microclimatic variation among plots.

Daily records from the Humán Agrometeorological Station of the Agromet-INIA network were used as a local ground-based reference to evaluate temporal consistency and to fill data gaps in the gridded series. Gap filling was performed only after verifying concordance between the local station and the gridded data during overlapping periods. This approach preserved the temporal structure of the climatic series and minimized inconsistencies associated with individual data sources.

Daily solar radiation data were obtained from Climate Engine, a cloud-based platform that integrates climate and remote-sensing datasets for environmental monitoring and natural resource applications [36], using the ERA5 climate dataset [37]. Potential evapotranspiration (PET) was estimated using these radiation data in conjunction with daily maximum and minimum air temperatures. Specifically, PET was calculated via the Hargreaves–Samani approach, a method appropriate when the comprehensive meteorological data required by more data-intensive methods are unavailable [38,39]. Daily PET values were aggregated to annual totals for each establishment year. Finally, daily vapor pressure deficit (VPD) was calculated from the maximum and minimum air temperatures following Allen et al. [40].

Annual precipitation (Pp), growing-season precipitation (PpGs), PET, VPD, frost days, and hot days were summarized for each establishment period. Following Álvarez et al. [9], PpGs was defined as the cumulative precipitation from September through April. PpGs was calculated for each of the three growing seasons following planting and subsequently averaged within each rotation. Frost days were defined as days with minimum temperatures below 0 °C, while hot days were defined as days with maximum temperatures above 35 °C. Annual values were averaged across each establishment period and subsequently used to compare the climatic context of the first and second rotations.

The climatic water balance was calculated using Equation (4):

*Water balance* = *Pp* − *PET* (4)

The aridity index (AI) was calculated using Equation (5):

*AI* = *Pp*/*PET* (5)

### 4.5. Data Analysis

Climatic variables were first summarized to compare environmental conditions during the establishment phase of the first and second rotations. Annual precipitation (Pp), growing-season precipitation (PpGs), PET, climatic water balance, aridity index (AI), VPD, frost days, and hot days were calculated for each year and subsequently averaged within each establishment period. Percentage changes between rotations were calculated relative to the first rotation. For climatic water balance, absolute differences were emphasized because values shifted from positive to negative between rotations.

Survival, RCD, height, and stand volume at age 3 were analyzed using repeated-measures mixed-effects models (PROC MIXED) in SAS version 9.4 (SAS Institute Inc., Cary, NC, USA). Rotation (R), soil preparation (S), fertilization (F), weed control (WC), and all their interactions were included as fixed effects. Block and the block × soil preparation interaction were included as random effects to account for the split-plot experimental structure, with soil preparation applied at the main-plot level. Because the same experimental units, treatment identities, and plot boundaries were maintained across rotations, rotation was treated as a repeated factor, with the plot specified as the repeated subject and an unstructured covariance matrix used to model correlations between observations from the same plot. Adjusted means for the eight treatment combinations were compared within each rotation and response variable using the LSMEANS statement with Tukey’s adjustment. Interactions involving rotation were used to determine whether treatment responses differed between establishment periods and were interpreted using the corresponding marginal means. Model assumptions were evaluated using residual diagnostics, including the Shapiro–Wilk (PROC UNIVARIATE) and Levene’s tests. Stand volume was log-transformed to satisfy model assumptions, although untransformed means are reported. Treatment effects and interactions were considered significant at α = 0.05. Rotation was interpreted as a comparison between the two observed establishment periods rather than as a randomized climatic treatment.

Treatment means were used to calculate absolute and relative responses to the control treatment (S0F0WC0) within each rotation, as described in Section 4.3. Rotation-to-rotation changes were calculated for each treatment combination by comparing second-rotation means with first-rotation means at age 3. Treatments were ranked based on stand volume within each rotation, with rank 1 representing the highest value. Finally, linear regression was used to evaluate the relationship between first- and second-rotation stand volume across the eight treatment combinations. The regression models were implemented using the PROC REG procedure with the NOINT option in SAS. The model was used to estimate the proportion of first-rotation stand volume maintained during the second rotation, with the slope interpreted as the rotation-to-rotation proportional response. The coefficient of determination (R^2^) and the significance of the fitted relationship were used to describe the association between first- and second-rotation treatment performance.

## 5. Conclusions

Early establishment performance during the second rotation of *P. radiata* was substantially lower than during the first, coinciding with lower precipitation, higher evaporative demand, and a shift from a positive to a negative climatic water balance. Although weed control mitigated some of these losses, none of the evaluated treatments restored first-rotation performance under the drier establishment conditions.

1. Weed control was the most influential treatment component. It was the only treatment that consistently improved survival and all growth variables during the second rotation, while treatments including weed control retained the highest stand-volume rankings across both rotations.
2. Water availability remained the dominant constraint on early stand performance. Subsoiling and fertilization alone were insufficient to offset the decline under second-rotation conditions, indicating that current establishment practices cannot fully compensate for substantially drier conditions.
3. The marked reduction in early stand volume demonstrates a substantial productivity gap between rotations. Continued monitoring is required to determine whether this gap persists through canopy closure and rotation age.

## Figures and Tables

**Table 1:** Comparison of climatic conditions during the establishment phase of the first (2000–2003) and second (2022–2025) *Pinus radiata* rotations on sandy soils in south-central Chile. Values represent means across the years included in each establishment period.

Variable	First Rotation (2000–2003)	Second Rotation (2022–2025)	Change (%)
Pp (mm year^−1^)	1337	898	−32.8
PpGs (mm growing-season^−1^)	508	288	−43.3
PET (mm year^−1^)	1077	1153	7.1
Water balance (mm year^−1^)	260	−255	—
Aridity index (Pp/PET)	1.25	0.78	−37.6
VPD (kPa)	0.71	0.81	14.1
Frost days (days year^−1^)	25	42	68.0
Hot days (days year^−1^)	2.3	6.3	173.9

Mean annual precipitation is denoted as Pp. Growing-season precipitation (PpGs) corresponds to cumulative precipitation from September through April. Potential evapotranspiration (PET) was estimated using the Hargreaves–Samani equation. Vapor pressure deficit is denoted as VPD. Water balance was calculated as precipitation minus PET (Pp−PET), and the aridity index (AI) as the ratio of Pp to PET. Frost days are defined as days with Tmin < 0 °C, and hot days as days with Tmax > 35 °C. Percent change was calculated relative to the first rotation.

**Table 2:** Treatment means, absolute responses, and relative responses versus the control (S0F0WC0) for diameter, height, stand volume, and survival at age 3 during the first and second rotations. Different lowercase letters indicate significant differences among treatment means within the same rotation and response variable (*p* < 0.05).

Rotation/ Treatment	Diameter			Height			Stand Volume			Survival
	Mean	Response		Mean	Response		Mean	Response		Mean
	cm	cm	%	m	m	%	m^3^ ha^−1^	m^3^ ha^−1^	%	%
First Rotation										
S0F0WC0	3.6 c			1.5 c			1.9 c			75.0 b
S0F0WC1	5.6 b	1.9	54.5	2.2 b	0.7	43.6	8.8 b	6.9	354.6	99.3 a
S0F1WC0	3.8 c	0.2	4.7	1.6 c	0.1	4.2	2.0 c	0.1	4.1	67.8 b
S0F1WC1	6.1 ab	2.5	68.5	2.3 ab	0.8	49.7	10.8 ab	8.9	456.3	98.3 a
S1F0WC0	3.8 c	0.2	5.6	1.6 c	0.1	1.9	2.7 c	0.8	41.5	93.3 a
S1F0WC1	5.7 b	2.1	56.7	2.3 b	0.7	47.8	9.3 b	7.4	381.5	99.0 a
S1F1WC0	4.1 c	0.5	12.8	1.7 c	0.2	9.8	3.4 c	1.5	76.3	92.3 a
S1F1WC1	6.5 a	2.9	80.9	2.5 a	0.9	63.4	13.8 a	11.9	612.3	99.5 a
Second Rotation										
S0F0WC0	2.4 bcd			1.0 de			0.3 bc			37.0 b
S0F0WC1	3.4 abc	0.9	37.0	1.3 abc	0.3	33.8	1.6 abc	1.3	460.4	83.0 a
S0F1WC0	2.5 bcd	0.1	0.5	1.1 bcde	0.1	6.9	0.4 bc	0.1	11.5	34.8 b
S0F1WC1	3.8 a	1.4	55.1	1.5 a	0.5	50.4	2.5 a	2.2	787.6	83.5 a
S1F0WC0	1.9 d	−0.5	−20.1	0.8 e	−0.2	−15.5	0.2 c	−0.1	−47.5	36.8 b
S1F0WC1	3.3 abc	0.8	34.4	1.3 abcd	0.3	30.4	1.8 abc	1.5	541.6	89.8 a
S1F1WC0	2.3 cd	−0.1	−5.5	1.0 cde	0.0	0.4	0.4 bc	0.1	40.6	46.3 b
S1F1WC1	3.6 ab	1.2	47.5	1.4 ab	0.4	45.5	2.2 ab	1.9	661.1	86.8 a

Treatment codes are defined as follows: soil preparation (S), where S0 = shovel planting and S1 = subsoiling + bedding; fertilization (F), where F0 = B-only and F1 = N, P, K, and B fertilization; and weed control (WC), where WC0 = baseline vegetation control only and WC1 = baseline vegetation control plus additional 2-year banded vegetation control. Pairwise comparisons were based on Tukey-adjusted comparisons following the mixed-effects analysis. Operational prescriptions, fertilizer doses, and herbicide products differed between rotations (see Section 4.2). Diameter corresponds to root collar diameter (RCD). Stand volume is expressed as cumulative volume per hectare. Absolute responses were calculated as the difference between the treatment mean and the control (S0F0WC0) mean within each rotation. Relative responses were calculated as a percentage: [(Treatment mean − S0F0WC0 mean)/S0F0WC0 mean] × 100. Survival is reported as a mean percentage.

**Table 3:** Summary of the statistical significance (*p*-values) for the effects of rotation (R), soil preparation (S), fertilization (F), weed control (WC), and their interactions on survival, diameter, height, and stand volume at age 3 during the first (2000–2003) and second (2022–2025) rotations. The first- and second-rotation rows represent rotation-specific simple-effects analyses obtained from the combined repeated-measures mixed-effects models, whereas the “Between rotations” rows represent interactions involving rotation. Values in bold indicate *p*-values < 0.05.

Rotation	Effect	Survival	Diameter	Height	Stand Volume
First	S	**0.013**	0.073	0.056	**0.041**
First	F	0.117	**0.002**	**0.001**	**0.002**
First	WC	**<0.001**	**<0.001**	**<0.001**	**<0.001**
First	S × F	0.162	0.359	0.160	0.132
First	S × WC	**<0.001**	0.934	0.263	0.495
First	F × WC	0.162	0.066	0.293	**0.009**
First	S × F × WC	0.384	0.582	0.521	0.346
Second	S	0.266	0.406	0.401	0.910
Second	F	0.751	0.069	**0.013**	0.197
Second	WC	**<0.001**	**<0.001**	**<0.001**	**<0.001**
Second	S × F	0.582	0.708	0.706	0.751
Second	S × WC	0.933	0.521	0.498	0.948
Second	F × WC	0.516	0.509	0.652	0.406
Second	S × F × WC	0.314	0.436	0.600	0.494
Between rotations	R	**<0.001**	**<0.001**	**<0.001**	**<0.001**
Between rotations	R × S	0.382	0.218	0.183	0.287
Between rotations	R × F	0.356	0.180	0.887	0.437
Between rotations	R × WC	**<0.001**	**<0.001**	**<0.001**	**<0.001**
Between rotations	R × S × F	0.972	0.635	0.441	0.549
Between rotations	R × S × WC	**0.011**	0.493	0.886	0.117
Between rotations	R × F × WC	0.235	0.268	0.718	0.516
Between rotations	R × S × F × WC	0.471	0.148	0.240	0.176

## Data Availability

Restrictions apply to the datasets. The datasets presented in this article are not readily available because the data are part of an ongoing study.

## Abbreviations

The following abbreviations are used in this manuscript:

AI	Aridity index
B	Boron
CR2MET	Center for Climate and Resilience Research Meteorological Dataset
DS	Dry sand
F	Fertilization
H	Total tree height
INIA	Instituto de Investigaciones Agropecuarias
K	Potassium
N	Nitrogen
P	Phosphorus
PET	Potential evapotranspiration
Pp	Precipitation
PpGs	Growing-season precipitation
R	Rotation
RCD	Root collar diameter
S	Soil preparation
VPD	Vapor pressure deficit
VI	Volume index
WC	Weed control

## References

[1] Rubilar R.A., Valverde J.C., Barrientos G., Campoe O.C. (2024). Water and temperature ecophysiological challenges of forests plantations under climate change. Forests.

[2] Allen C.D., Breshears D.D., McDowell N.G. (2015). On underestimation of global vulnerability to tree mortality and forest die-off from hotter drought in the Anthropocene. Ecosphere.

[3] Wilmking M., van der Maaten-Theunissen M., van der Maaten E., Scharnweber T., Buras A., Biermann C., Gurskaya M., Hallinger M., Lange J., Shetti R. (2020). Global assessment of relationships between climate and tree growth. Glob. Change Biol..

[4] IPCC (2022). Climate Change 2022: Impacts, Adaptation and Vulnerability.

[5] Rubilar R., Bozo D., Albaugh T., Cook R., Campoe O., Carter D., Allen H.L., Álvarez J., Pincheira M., Zapata Á. (2023). Rotation-age effects of subsoiling, fertilization, and weed control on radiata pine growth at sites with contrasting soil physical, nutrient, and water limitations. For. Ecol. Manag..

[6] Vargas F., Gonzalez-Benecke C.A., Ahumada R. (2022). Long-term responses to competing vegetation management for *Pinus radiata*. Forests.

[7] Rubilar R.A., Lee Allen H., Fox T.R., Cook R.L., Albaugh T.J., Campoe O.C. (2018). Advances in silviculture of intensively managed plantations. Curr. For. Rep..

[8] Mead D.J. (2013). Sustainable Management of Pinus radiata Plantations.

[9] Alvarez J., Allen H.L., Albaugh T.J., Stape J.L., Bullock B.P., Song C. (2013). Factors influencing the growth of radiata pine plantations in Chile. Forestry.

[10] Ojeda H., Rubilar R.A., Montes C., Cancino J., Espinosa M. (2018). Leaf area and growth of Chilean radiata pine plantations after thinning across a water stress gradient. N. Z. J. For. Sci..

[11] Watt M.S., Rolando C.A., Kimberley M.O., Coker G.W.R., Freckleton R. (2015). Using the age shift method to determine gains from weed management for *Pinus radiata* in New Zealand. Weed Res..

[12] Smethurst P.J., Nambiar E.K.S. (1989). Role of weeds in the management of nitrogen in a young *Pinus radiata* plantation. New For..

[13] Fox T.R. (2000). Sustained productivity in intensively managed forest plantations. For. Ecol. Manag..

[14] Zhang X.-Q., Kirschbaum M.U.F., Hou Z., Guo Z. (2004). Carbon stock changes in successive rotations of Chinese fir (Cunninghamia lanceolata (lamb) hook) plantations. For. Ecol. Manag..

[15] Huong V.D., Nambiar E.K.S., Hai N.X., Ha K.M., Dang N.V. (2020). Sustainable management of Acacia auriculiformis plantations for wood production over four successive rotations in South Vietnam. Forests.

[16] Hogan J.A., Domke G.M., Zhu K., Johnson D.J., Lichstein J.W. (2024). Climate change determines the sign of productivity trends in US forests. Proc. Natl. Acad. Sci. USA.

[17] Mekarni L., Cochard H., Grossiord C. (2025). Acclimation to high vapor pressure deficit in warmer air can reduce tree vulnerability to drought-induced mortality. Plant Cell Environ..

[18] Allen C.D., Macalady A.K., Chenchouni H., Bachelet D., McDowell N., Vennetier M., Kitzberger T., Rigling A., Breshears D.D., Hogg E.H. (2010). A global overview of drought and heat-induced tree mortality reveals emerging climate change risks for forests. For. Ecol. Manag..

[19] Ciais P., Reichstein M., Viovy N., Granier A., Ogée J., Allard V., Aubinet M., Buchmann N., Bernhofer C., Carrara A. (2005). Europe-wide reduction in primary productivity caused by the heat and drought in 2003. Nature.

[20] Carrasco G., Almeida A.C., Falvey M., Olmedo G.F., Taylor P., Santibanez F., Coops N.C. (2022). Effects of climate change on forest plantation productivity in Chile. Glob. Change Biol..

[21] Rubilar R.A., Albaugh T.J., Allen H.L., Alvarez J., Fox T.R., Stape J.L. (2013). Foliage development and leaf area duration in *Pinus radiata*. For. Ecol. Manag..

[22] Basso A.S., Miguez F.E., Laird D.A., Horton R., Westgate M. (2012). Assessing potential of biochar for increasing water-holding capacity of sandy soils. GCB Bioenergy.

[23] Dinamarca D.I., Galleguillos M., Seguel O., Faundez Urbina C. (2023). CLSoilMaps: A national soil gridded database of physical and hydraulic soil properties for Chile. Sci. Data.

[24] Qi J., Markewitz D., McGuire M.A., Samuelson L., Ward E. (2020). Throughfall reduction × fertilization: Deep soil water usage in a clay rich ultisol under loblolly pine in the Southeast USA. Front. For. Glob. Change.

[25] Gwaze D., Johanson M., Hauser C. (2007). Long-term soil and shortleaf pine responses to site preparation ripping. New For..

[26] Zhao D., Kane M., Borders B., Harrison M. (2009). Long-term effects of site preparation treatments, complete competition control, and repeated fertilization on growth of slash pine plantations in the Flatwoods of the Southeastern United States. For. Sci..

[27] Mason E.G., Skinner M.F., Barrow N.J., Lowe A., Bown H.E. (2010). Predicting phosphorus requirements of young *Pinus radiata* using sequential Bray soil extraction. Plant Soil.

[28] Cassiano C.C., Moreira R.M.e., Ferraz S.F.d.B. (2023). Fast-growing forest management to regulate the balance between wood production and water supply. Sci. Agric..

[29] Jones A.G., Cridge A., Fraser S., Holt L., Klinger S., McGregor K.F., Paul T., Payn T., Scott M.B., Yao R.T. (2023). Transitional forestry in New Zealand: Re-evaluating the design and management of forest systems through the lens of forest purpose. Biol. Rev. Camb. Philos. Soc..

[30] Garreaud R.D., Boisier J.P., Rondanelli R., Montecinos A., Sepúlveda H.H., Veloso-Aguila D. (2020). The Central Chile Mega Drought (2010–2018): A climate dynamics perspective. Int. J. Climatol..

[31] Araya-Osses D., Casanueva A., Román-Figueroa C., Uribe J.M., Paneque M. (2020). Climate change projections of temperature and precipitation in Chile based on statistical downscaling. Clim. Dyn..

[32] CIREN (1999). Soil Descriptions, Materials, and Symbols: Agrological Study of the VIII Region.

[33] Stolpe N. (2006). Descriptions of the Principal Soils of the VIII Region of Chile.

[34] Schlatter J.S., Gerding V. (1985). Boron deficiency in *Pinus radiata* D. Don plantations in Chile. II. Main causes and correction. Bosque.

[35] Alvarez-Garreton C., Mendoza P.A., Boisier J.P., Addor N., Galleguillos M., Zambrano-Bigiarini M., Lara A., Puelma C., Cortes G., Garreaud R. (2018). The CAMELS-CL dataset: Catchment attributes and meteorology for large sample studies—Chile dataset. Hydrol. Earth Syst. Sci..

[36] Huntington J.L., Hegewisch K.C., Daudert B., Morton C.G., Abatzoglou J.T., McEvoy D.J., Erickson T. (2017). Climate Engine: Cloud computing and visualization of climate and remote sensing data for advanced natural resource monitoring and process understanding. Bull. Am. Meteorol. Soc..

[37] Hersbach H., Bell B., Berrisford P., Hirahara S., Horányi A., Muñoz-Sabater J., Nicolas J., Peubey C., Radu R., Schepers D. (2020). The ERA5 global reanalysis. Q. J. R. Meteorol. Soc..

[38] Hargreaves G., Samani Z. (1985). Reference crop evapotranspiration from temperature. Appl. Eng. Agric..

[39] Ravazzani G., Corbari C., Morella S., Gianoli P., Mancini M. (2012). Modified Hargreaves-Samani equation for the assessment of reference evapotranspiration in Alpine River basins. J. Irrig. Drain. Eng..

[40] Allen R., Pereira L., Raes D., Smith M. (1998). FAO Irrigation and Drainage Paper No. 56.

